# Impact of government subsidy reforms on primary health care efficiency in rural eastern China: an interrupted time series analysis

**DOI:** 10.1186/s12913-025-13967-0

**Published:** 2026-01-07

**Authors:** Xiaoying Pu, Rong Liu, Zhuangfei Wang, Ting Huang, Xiaohe Wang, Yaming Gu

**Affiliations:** 1 School of Basic Medical Sciences, Hangzhou Normal University, Hangzhou, Zhejiang 311121 China; 2 School of Public Health, Hangzhou Medical College, Hangzhou, Zhejiang 311399 China; 3 Department of Health Reform, Zhejiang Provincial Health Commission, Hangzhou, Zhejiang 310006 China; 4 Shengzhou Traditional Chinese Medicine Hospital, Shaoxing, Zhejiang 312400 China; 5 School of Public Health, Hangzhou Normal University, Hangzhou, Zhejiang 311121 China

**Keywords:** Primary health care, Payment reform, Relative-value units, Rural health systems, Interrupted time series, China

## Abstract

**Background:**

Despite rising government subsidies, inefficiencies persist in China’s primary health-care system. We evaluated an eastern rural pilot that shifted subsidies from passive grants to strategic purchasing via fixed salaries plus activity-based payments using resource-based relative value units (RVUs).

**Methods:**

Interrupted time series (January 2015 - December 2022) assess the instantaneous and long-term effects of the reform (implemented January 2017) on monthly RVUs per employee across all primary health institutions (PHIs) and three sub-categories (central town/street PHIs, other PHIs, remote countryside PHIs). COVID-19 onset (January 2020) was incorporated as a secondary intervention to control for its confounding effect.

**Results:**

Post-reform, RVUs per employee were associated with a 61.17% level increase and a sustained upward slope (+3.13; 95% CI 1.65–4.61).The post-COVID slope was associated with a deceleration of −1.74 RVUs per month (95% CI:−3.23 to −0.24), consistent with pandemic disruption rather than definitive causation. Remote countryside PHIs showed no immediate level change (*p* = 0.18) but exhibited a significantly steeper post-reform slope (β₃ = +7.58; 95% CI: 3.42–11.75) and the steepest post-pandemic deceleration (slope change: −9.35; 95% CI:−12.14 to −6.55), indicating slope-based responsiveness. Cross-group comparisons suggest that smaller PHIs with higher subsidy dependence were more responsive to both the reform and the pandemic’s disruptive effects, although this association does not establish causation.

**Conclusions:**

Strategic purchasing was associated with moderate PHC efficiency gains in rural eastern China. Smaller, remote institutions demonstrated greater slope-based responsiveness to policy changes yet remained more vulnerable in terms of slope to external shocks such as COVID-19. Long-term efficiency gains require targeted support for underserved PHIs and continued refinement of activity-based payment mechanisms.

**Supplementary information:**

The online version contains supplementary material available at 10.1186/s12913-025-13967-0.

## Introduction

Primary health care (PHC) is the bedrock of resilient health systems and a prerequisite for universal health coverage [1–3]. In high-income settings, blended payment models—capitation plus fee-for-service—have expanded service volumes, yet often at the cost of supplier-induced demand and quality skimping [4–6]. In low- and middle-income countries (LMICs), fragmented financing, weak regulatory capacity and razor-thin budgets make output-based purchasing even harder, especially for preventive and public-health services [7–10].

China’s 2009 health-care reform abolished drug mark-ups and shifted primary-care providers to government subsidies, yet allocation remains largely passive and inefficiencies persist—especially in rural counties [10]. This creates a natural experiment to test whether strategic purchasing—linking subsidies to activity-based relative value units (RVUs)—can improve PHC efficiency without sacrificing fiscal sustainability. The quest for an “optimal” government subsidy mechanism is inherently context-specific. In China, subsidies to primary health institutions (PHIs; township and sub-district health centers) jumped from ¥19.3 billion in 2008 to ¥258.8 billion in 2021, which now constitute a major portion of their revenue, now averaging 43% of total revenue and > 60% in some provinces [11, 12]. Despite this massive investment, efficiency gains have been uneven, underscoring the need for nuanced, locally aligned payment designs that convert public spending into sustainable, high-quality services [12–14].

In response to the aforementioned challenges, previous research has delineated a comprehensive reform framework and spearheaded a pilot investigation [15, 16]. This initiative was designed to scrutinize and potentially revamp the existing government subsidy mechanisms. Central to this reform was the consolidation of the government subsidy allocated for the National Essential Public Health Service Package (NEPHSP) alongside a special subsidy designed to offset the financial impact of the zero-drug mark-ups policy [13, 16]. The NEPHSP subsidy is meticulously itemized according to a per capita budgeting approach; however, its structure does not establish a clear link with the performance metrics of primary care physicians. These physicians, who are predominantly salaried and driven by quotas set by Chinese authorities [10]. The special subsidy, intended to resolve financial shortfalls from zero-drug make-ups [12, 13], may paradoxically create a system where the pursuit of efficiency and performance is neither incentivized nor recognized, which could ultimately impede the enthusiasm of PHIs to provide high-quality healthcare services.

The integrated subsidies above were converted into blended payments, with the funding structure illustrated in Pu et al. [16]. Under this arrangement, the incoming subsidy is split into two blocks: (1) an input-based guaranteed block (≈50% of the total subsidy) covering professional basic salaries, social-security contributions, and remote-area allowances, paid irrespective of service volume; and (2) an activity-based variable block (≈50% of the total subsidy) whereby all services are scored using RBRVS weights, summed to monthly RVUs, and multiplied by a conversion factor (≈CNY 11 per RVU) to form the variable payroll pool, which is then distributed to individual staff pro-rata to their documented RVUs. The reform introduced a specialized IT system to integrate and evaluate RBRVS data, ensuring that government subsidies are effectively aligned with the performance metrics of healthcare providers. Additionally, subsidies for infrastructure, medical equipment, and IT expenditures remain item budgeted.

Despite an initial assessment by 2018, the pilot reform’s impact on PHC efficiency lacked the depth of robust design and rigorous statistical analysis [16]. The extent to which the efficiency of PHC has been enhanced remains uncertain, giving the fact their performance is important for health system success [17]. Therefore, the primary objective of this study is to conduct a thorough re-evaluation of the reform, with a particular emphasis on its impact on efficiency. The findings and lessons learned from this study aim to inform and guide future policy adjustments and optimizations, while also providing valuable insights for LMICs contemplating analogous government subsidy reforms in primary care.

## Materials and methods

### Study design

Shengzhou County (≈730,000 residents) comprises 21 township-level PHIs. We adopted the official three-tier stratification published in Joint Circular No. [2016]176 (Shenzhou Health Bureau & Finance Bureau), which assigns each PHI a Geographic Practice Cost Index (GPCI) reflecting remoteness, population size and historical running costs. The GPCI was used to weight RVUs of each PHI [15], was frozen at baseline (1 January 2017) and used solely for stratification. Consequently, the 21 PHIs fall into three mutually exclusive groups: central town and street PHIs (*n* = 9, GPCI = 1.0), remote countryside PHIs (*n* = 6, GPCI = 1.5), and others (*n* = 6, GPCI = 1.2).

This study employed an interrupted time series analysis (ITSA), both in single- and multi-group formats, to assess the impact of the government subsidy reform on the efficiency of PHIs. ITSA is a robust method recognized for evaluating the longitudinal impacts of interventions, and its application in this study is particularly pertinent given the concurrent COVID-19 pandemic’s influence, which served as an additional natural experiment intervention. The single-group ITSA was utilized to compare the levels and trends before and after each intervention, thereby evaluating the comprehensive effect on all PHIs as a whole and the individual effects on each sub-category of PHIs. Because no external county could provide monthly RVU panels, we used the three PHI strata as mutual controls, following prior ITSA studies where randomization or external controls are infeasible [18]. Parallel trends were verified including level difference and trend difference between three PHI sub-groups before the reform.

Given the COVID-19 pandemic’s significant influence on PHIs, it was incorporated as an intervention factor within the research model to control for its confounding effects [19]. Internal controls were implemented to account for potential concurrent confounders by comparing different categories of groups. Efficiency was quantified by the monthly RVUs per employee. Although RVUs do not capture clinical quality, they constitute a longitudinal, institution-level output metric. Internationally, RVU-based productivity is a validated efficiency indicator [16, 20, 21]. The hypotheses under investigation were:

- The pilot reform will result in a significant enhancement in PHI efficiency.
- The change in efficiency will vary across different PHI categories due to their inherent heterogeneity.
- Larger PHIs are expected to show greater adaptability to the reform, while smaller institutions may be more vulnerable.

### Data collection

The study spanned 96 months (January 2015–December 2022), covering the simulated reform period (January 2015–December 2016), its official launch in January 2017, and the onset of COVID-19 in January 2020. Pre-reform (2015–2016) monetary and RVU values were derived from a static micro-simulation model prepared under Joint Circular No. [2016]176 (Shenzhou Health Bureau & Finance Bureau). Data were sourced through two primary methods. Initially, annual reports from the National Health Financial Annual Reports of 2015–2022 provided detailed yearly information on PHIs’ basic details, income, and expenditures. This data included general income and expenditure tables, tables detailing revenue and expenditures from government subsidies, and basic figures and financial analysis. Secondly, monthly RBRVS payment data were procured from the specialized IT system established for the reform [16]. The Resource-Based Relative Value Scale (RBRVS) is a weighting system that assigns a RVU to each service according to the time, technical intensity, and practice cost required to deliver it [21]. The RVUs catalogue of NEPHSP and complementary compensation for medical services are shown in the Appendix 1, including items, service contents, data source, statistical definition, and quality control. However, it should be noted that RVUs reflect service volume and intensity, not clinical quality, patient outcomes, or cost-effectiveness.

### Statistical analysis

**Fig. 1:**
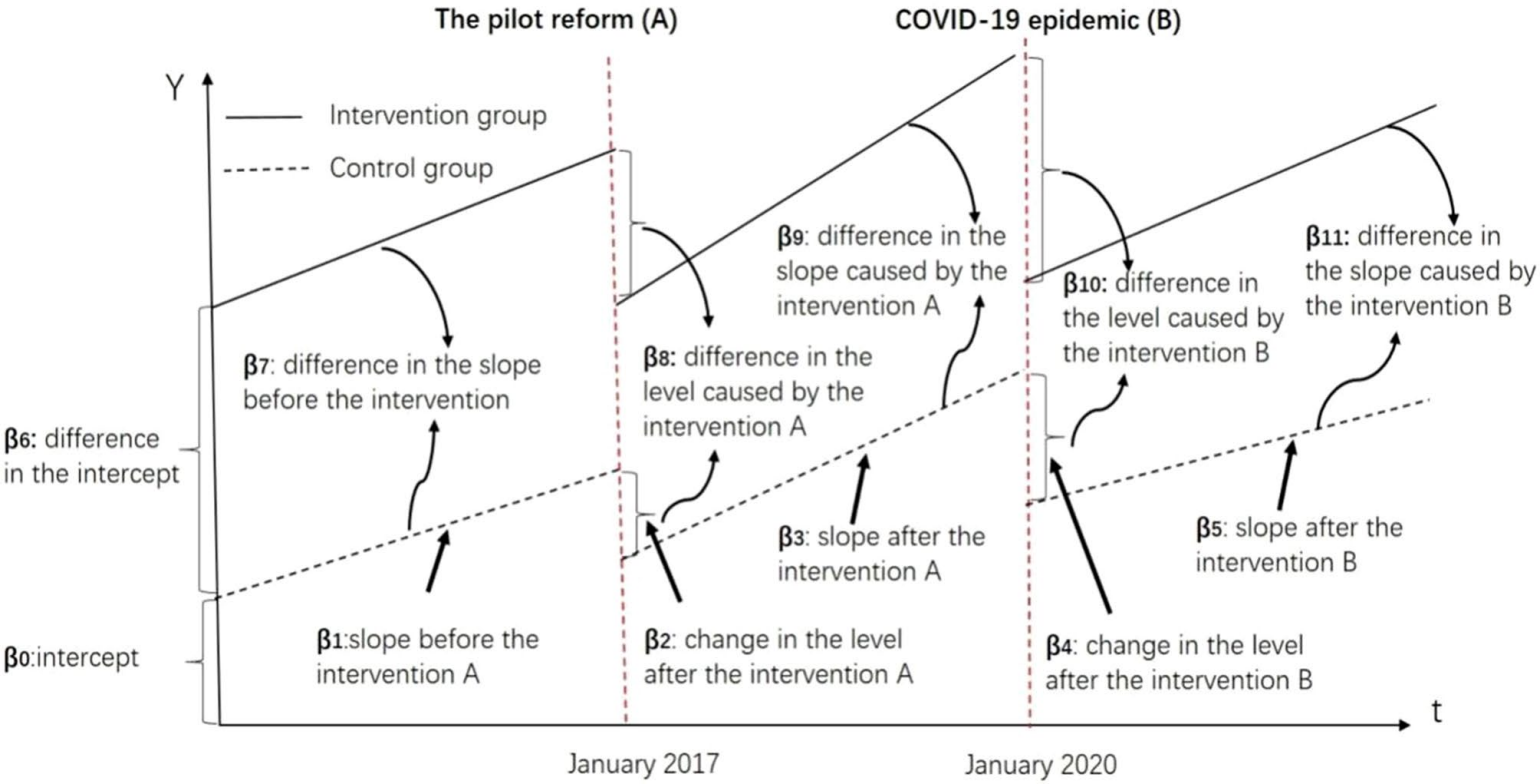
Visual description of multi-group ITSA

Descriptive statistics were employed to summarize the pre- and post-intervention income, expenditures, and monthly RVUs per employee. Subsequently, segmented linear regression models were applied to conduct both single- and multi-group ITSAs, probing the impact of the interventions. Figure 1 provides a visual representation of multi-group ITSA, and the models for single- (1) and multi-group (2) ITSA are as follows:

1 $$\begin{aligned}Y_t&=\beta_0+\beta_1T_t+\beta_2X_A+\beta_3X_A(T_t-24)\cr&\quad+\beta_4X_B+\beta_5X_B(T_t-60)+\varepsilon_t \end{aligned}$$

2 $$\begin{aligned}Y_t&=\beta_0+\beta_1T_t+\beta_2X_A+\beta_3X_A(T_t-24)\cr&\quad+\beta_4X_B+\beta_5X_B(T_t-60)+\beta_6Z\cr&\quad+\beta_7ZT_t+\beta_8ZX_A+\beta_9ZX_A(T_t-24)\cr&\quad+\beta_10ZX_B+\beta_11ZX_B(T_t-60)+\varepsilon_t\end{aligned}$$

In these equations, Y_t_ denotes the RVUs per employee for month t, T_t_ represents the time in months since the observation commencement. X_A_ and X_B_ are binary variables signifying interventions A and B, respectively (0 for pre-intervention periods; 1 otherwise), with Z distinguishing between treatment and control groups (0 for control, 1 for treatment). “β_8_” represents the difference in the average level of monthly RVUs per employee between the intervention and control periods (reflecting the instantaneous effect of the reform), and “β_9_” indicates the difference in slope between the intervention and control periods (indicating the long-term effect of the reform). Similarly, “β_10_” and “β_11_” represent the differences in the level and slope caused by the pandemic between the intervention and control periods.

The Durbin-Watson test was utilized to assess residual autocorrelations, and the Cumby-Huizinga general tests (via the actest function in Stata) were employed to determine the appropriate lag for auto-correlation [18, 22]. The results of these tests are detailed in Appendix 2. The Newey-West method was applied to address heteroscedasticity and autocorrelation when necessary [22], with the seasonal pattern adjusted using Fourier terms [18, 22]. All statistical analyses were performed using Stata 18.0. The complete do-file are shown in the Appendix 3.

## Results

### Restructuring subsidies and efficiency

A comprehensive summary of the findings is provided in Table 1. Prior to the reform, basic salaries and RBRVS payments constituted 31.10% and 36.28% of the total government subsidy, respectively. Post-reform, these proportions minimally shifted to 30.50% and 36.25%. Significantly, the reform catalyzed a considerable augmentation in total RBRVS, RVUs per employee, and average salary, with total RVUs increasing by 49.22% from 3850.6 thousand to 5745.9 thousand, monthly RVUs per employee soaring by 61.17% from 2526.7 to 4072.2, and the average salary escalating by 43.41% from ¥87.4 thousand to ¥125.4 thousand. However, it is imperative to highlight that the growth rates of these three indicators experienced a marked deceleration following the pandemic, with total RVUs witnessing a 15.35% rise, RVUs per employee increasing by 15.95%, and the average salary’s growth rate moderating to 10.64%.

**Table 1:** The reformed structure of government subsidies for primary health institutions befor and after the reform

Items	Before reform* (24 months)			After the pilot reform (36 months)				After COVID-19 pandemic (36 months)			
	2015	2016	Mean	2017	2018	2019	Mean	2020	2021	2022	Mean
Total revenue (¥ million) (1)	360.80	400.47	380.64	479.62	528.79	581.78	530.06	550.12	590.87	689.72	610.24
Total expenditure (¥ million) (2)	364.13	415.76	389.95	474.35	510.46	564.98	516.60	541.73	570.69	673.57	595.33
Net income(¥million) (3) = (1) - (2)	−3.33	−15.29	−9.31	5.27	18.33	16.81	13.47	8.39	17.21	18.00	14.53
Total financial subsidy (¥ million) (4)	113.26	114.81	114.04	153.26	172.9	176.65	167.60	176.05	175.12	242.46	197.88
#NEPHSP subsidy (5)	32.21	34.12	33.17	36.83	43.40	45.86	42.03	46.71	52.66	55.00	51.46
#Subsidy covering expenditure–revenue deficits (6)	30.27	57.08	43.68	65.78	67.45	76.33	69.85	81.03	82.65	84.95	82.88
Sum of reform (7) = (5) + (6) = (8) + (10)	62.48	91.20	76.84	102.61	110.85	122.19	111.88	127.74	132.52	139.95	133.40
# Basic salaries (¥ million) (8)	23.96	46.98	35.47	51.41	50.23	51.74	51.13	56.21	56.57	62.49	58.42
As % of total financial subsidy (9) = (8)/(4) × 100%	21.15	40.92	31.10	33.54	29.05	29.29	30.50	31.93	32.30	25.77	29.53
#RBRVS payments (¥ million) (10)	38.52	44.22	41.37	51.20	60.62	70.45	60.76	71.53	72.95	74.47	72.98
As % of total financial subsidy(11) = (10)/(4) × 100%	34.01	38.52	36.28	33.41	35.06	39.88	36.25	40.63	41.66	30.71	36.88
Total RBRVS (thousand RVUs) (12)	3266.7	4434.6	3850.6	5299.3	5805.4	6133.1	5745.9	6306.7	6741.5	6834.8	6627.7
Total employees (13)	1509	1539	1524	1484	1381	1368	1411	1356	1409	1446	1404
RVUs per employee (14) = (12)/(13)	2164.8	2881.5	2526.7	3571.0	4203.8	4483.3	4072.2	4651.0	4784.6	4726.7	4721.7
Average salary per employee (¥ thousand) (15)	78.6	96.1	87.4	119.6	129.7	127.2	125.4	127.5	130.9	156.8	138.7

Note: *Pre-reform (2015–2016) monetary and RVU values were generated by a static micro-simulation model; RVUs = relative-value units

Compared with the pre-reform block-grant baseline (2015–2016), the county channeled an additional CNY 19.4 million annually (2017–2019) into activity-based RBRVS payments, yielding 1.83 million extra RVUs each year. The resulting incremental cost-RVU ratio (ICRR) averaged 10.6 CNY per additional RVU (95% CI 9.1–12.2). Remote PHIs delivered the highest fiscal efficiency—8.1 CNY per RVU (95% CI 6.4–9.9)—whereas central-town PHIs required 13.4 CNY (95% CI 11.0–15.8), demonstrating markedly better value for money in the most underserved areas.

We also examined capacity and subsidy dependence across PHI categories. Mean employee numbers in 2015 differed markedly: 136.3 (SD 55.4) in central town/street PHIs, 39.5 (SD 11.6) in other PHIs and 10.1 (SD 5.3) in remote countryside PHIs (all pairwise *p* < 0.001). Meanwhile, the share of government subsidies in total revenue rose as size fell: 33.25% for central town/street PHIs, 42.53% for other PHIs, and 55.87% for remote countryside PHIs (pairwise χ^2^ tests, all *p* < 0.001), suggesting that smaller PHIs are more subsidy-dependent. Detailed staff numbers and financial subsidies by PHI type before and after the reform are provided in Appendix 4.

### Single-group ITSA: efficiency changes

**Fig. 2:**
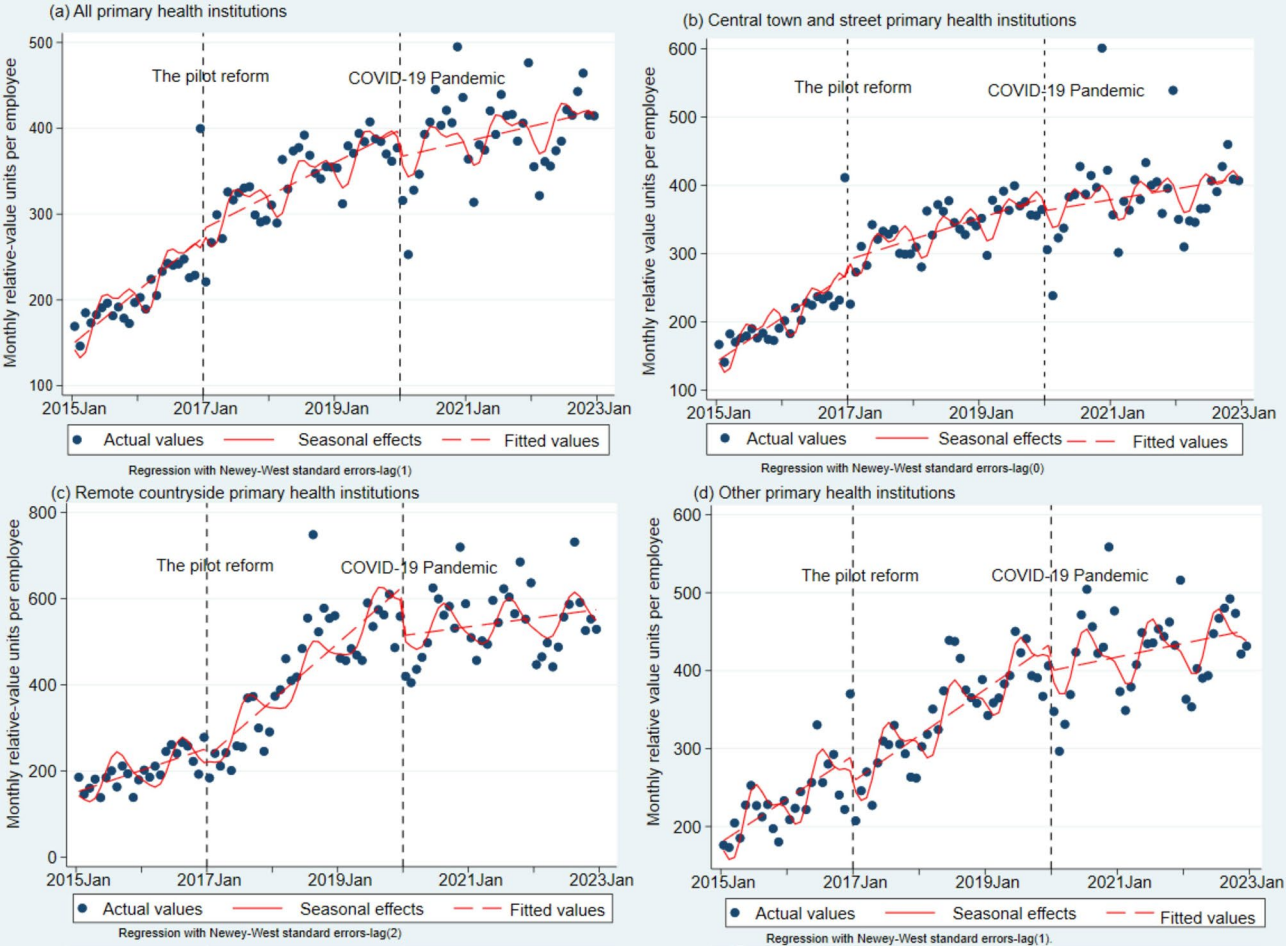
Single-group interrupted time series analysis of monthly relative-value units per employee

The single-group ITSA, which examines the monthly RVUs per employee, is detailed in Table 2 and Fig. 2. A discernible growth slope (β_1_ = 4.40; 95%CI: 2.40–6.40) over a 24-month pre-reform period was observed. This growth slope varied among different PHI categories (Fig. 2b–d), being statistically significant in central town and street PHIs (β_1_ = 4.40; 95%CI: 1.86–6.93) and other PHIs (β_1_ = 3.79; 95%CI: 1.59–5.98), yet non-significant in remote countryside PHIs (β_1_ = 2.83, *p* = 0.138). The reform’s implementation sustained a statistically significant growth slope in PHIs (β_1_ + β_3_ = 2.86; 95%CI: 1.98–3.74) over the subsequent 60 months. However, the post-reform level and trend changes did not reach statistical significance (β_2_ = 25.20, *p* = 0.175; β_3_ = −1.55, *p* = 0.174). In terms of post-reform heterogeneity, the growth slope in remote countryside PHIs markedly increased from 2.83 to 11.04 (trend change, β_3_ = 7.58; 95%CI: 3.42–11.75), whereas the other categories exhibited no significant trend (trend changes, β_3_ = −2.29 and 0.76, respectively; both *p* > 0.05). The level change post-reform was also not statistically significant across any category (level changes, β_2_ = 38.82, −15.92, and 10.72; all *p* > 0.05). Following the pandemic, the growth slope diminished (β_5_ = −1.74; 95%CI: −3.23 to −0.24), although the corresponding level change remained non-significant (β_4_ = −19.55, *p* = 0.231). In terms of post-pandemic heterogeneity (see Table 2), remote countryside PHIs experienced the most rapid deceleration in both level and trend changes (β_4_ = −96.14 [95%CI: −155.90 to −36.39]; β_5_ = −9.35 [95%CI: −12.14 to −6.55]), while central town and street PHIs demonstrated no significant alterations (β_4_ = −4.40, *p* = 0.832; β_5_ = −1.19, *p* = 0.219).

**Table 2:** Single-group interrupted time series analysis of monthly relative-value units per employee

Periods	Parameters	All PHIs (95% CI)	Central town and street PHIs (95% CI)	Remote countryside PHIs (95% CI)	Other PHIs (95% CI)
Period I (Before the pilot reform#, n = 24 months)	Baseline level (β_0_)	159.61 (132.94–186.27)^***^	150.94 (114.90–186.98)^***^	166.32 (113.28–219.37)^***^	187.80 (156.62–218.99)^***^
	Baseline trend (β_1_)	4.40 (2.40–6.40)^***^	4.40 (1.86–6.93)^***^	2.83 (−0.91–6.56)	3.79 (1.59–5.98)^***^
Period II (After the pilot reform, n = 36 months)	Level change after reform (β_2_)	25.20 (−11.22–61.60)	38.82 (−7.38–85.01)	10.72 (−57.28–78.70)	−15.92 (−55.88–24.05)
	Trend change after reform (β_3_)	−1.55 (−3.78–0.68)	−2.29 (−5.12–0.54)	7.58 (3.42–11.75)^***^	0.76 (−1.69–3.21)
	Trend after reform (β_1 +_ β_3_)	2.86 (1.98–3.74)^***^	2.53 (1.61–3.45)^***^	11.04 (8.57–13.51)^***^	4.93 (3.62–6.24)^***^
Period III (After COVID-19 pandemic, *n* = 36 months)	Level change after the pandemic (β_4_)	−19.55 (−51.55–12.45)	−4.40 (−45.00–36.20)	−96.14 (−155.90–−36.39)^**^	−23.21 (−58.34–11.91)
	Trend change after the pandemic (β_5_)	−1.74 (−3.23 to −0.24)^*^	−1.19 (−3.09–0.71)	−9.35 (−12.14 to −6.55)^***^	−3.472 (−5.116 to −1.828)^***^
	Trend after pandemic (β_1 +_ β_3 +_ β_5_)	1.12 (−0.47–2.71)	1.34 (−0.53–3.20)	1.69 (−1.76–5.13)	1.46 (−0.86–3.77)

Note: ^*^ *p* < 0.05, ^**^p < 0.01, ^***^ *p* < 0.001; #Pre-reform (2015–2016) monetary and RVU values were generated by a static micro-simulation model; seasonal pattern was adjusted by fitting Fourier terms in the regression model by two sine/cosine pairs

### Multi-group ITSA: comparative efficiency impact

**Fig. 3:**
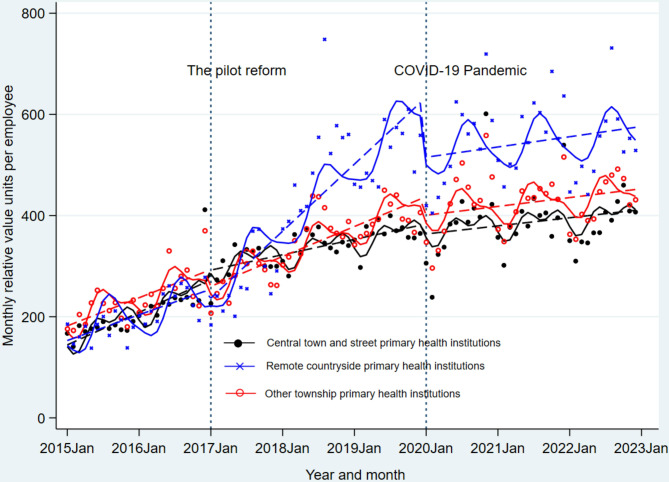
Multi-group interrupted time-series analysis of monthly relative-value units per employee. Note: the dots indicate actual values, the dashed lines indicate the model-fitted values and the solid lines indicate seasonal effects

The multi-group ITSA results, presented in Table 3 and Fig. 3, reveal that prior to the reform, no statistically significant differences in trends were identified between any pair of the three PHI categories, including central town and street versus remote countryside (β_7_ = 1.12, *p* = 0.507), central town and street versus others (β_7_ = 0.72, *p* = 0.696), and others versus remote countryside (β_7_ = 0.40, *p* = 0.765). Similarly, level differences were not statistically significant between central town and street versus remote countryside (β_6_ = 8.64, *p* = 0.563) and others versus remote countryside (β_6_ = 28.52, *p* = 0.062), indicating comparability before the reform. Post-reform, all three groups exhibited statistically significant monthly growth trends (β_1_ + β_3_ = 2.53, 11.04, and 4.93, respectively; all *p* < 0.001), with inter-group differences widening (Fig. 2). The central town and street PHIs demonstrated a slower growth slope (β_1_ + β_3_ + β_7_ + β_9_ = 2.53 RVUs/month; 95%CI: 1.55–3.52) compared to other PHIs (4.93 RVUs/month; 95%CI: 3.64–6.21), which in turn was slower than that of remote countryside PHIs (11.04 RVUs/month; 95%CI: 8.82–13.25). Following the pandemic, the multi-group ITSA revealed that the growth slope of central town and street PHIs was more rapid than that of remote countryside PHIs (trend difference, β_7_ + β_11_ = 9.27; 95%CI: 3.77–14.78), and the latter’s slope was slower than other PHIs (trend difference, β_7_ + β_11_ = −6.27; 95%CI: −11.58 to −0.97). Considering the combined impact of the reform and the pandemic, the trend difference returned toward baseline, with no significant differences in trends between any pair of the three groups (see period III in Table 3).

**Table 3:** Multi-group interrupted time series analysis of monthly relative-value units per employee

Periods/Parameters	Central town and street PHIs vs. Remote countryside PHIs	Other PHIs vs. Remote countryside PHIs	Central town and street PHIs vs. Other PHIs
**Period I (Before the pilot reform, n = 24 months)**			
Trend of control (β_1_)	4.24 (2.69–5.78)^***^	4.24 (2.69–5.78)^***^	4.64 (2.50–6.77)^***^
Level difference (treatment - control, β_6_)	8.64 (−37.96–20.66)	28.52 (−1.45–58.50)	−37.57 (−70.52 to −4.61) ^*^
Trend difference (treatment - control, β_7_)	1.12 (−2.21 to 4.45)	0.40 (−2.24–3.03)	0.72 (−2.92–4.36)
**Period II (After the pilot reform, n = 36 months)**			
Trend of control (β_1 +_ β_3_)	11.04 (8.82–13.25)^***^	11.04 (8.82–13.25)^***^	4.93 (3.64–6.21)^***^
Trend of treatment (β_1_ + β_3_ + β_7_ + β_9_)	2.53 (1.55–3.52)^***^	4.93 (3.64–6.21)^***^	2.53 (1.55–3.52)^***^
Trend difference (treatment - control, β_7_ + β_9_)	−8.50 (−10.93 to −6.08)^***^	−6.11 (−8.67–−3.55)^***^	−2.40 (−4.02 to −0.78)^**^
**Period III (After COVID-19 pandemic, n = 36 months)**			
Trend of control (β_1_ + β_5_)	−5.11 (−9.27 to −0.95)^*^	−5.11 (−9.27 to −0.95)^*^	1.16 (−2.13–4.45)
Trend of treatment (β_1_ + β_5_ + β_7_ + β_11_)	4.16 (0.56–7.77)^*^	1.16 (−2.13–4.45)	4.16 (0.56–7.77)^*^
Trend difference (treatment - control, β_7_ + β_11_)	9.27 (3.77–14.78)^**^	6.27 (0.97–11.58)^*^	3.00 (−1.88–7.88)
**Total impact**			
Trend of control (β_1_ + β_3_ + β_5_)	1.69 (−1.41–4.79)	1.69 (−1.41–4.79)	1.46 (−0.82–3.73)
Trend of treatment (β_1_ + β_3_ + β_5_ + β_7_ + β_9_ + β_11_)	1.34 (−0.88–3.56)	1.46 (−0.82–3.73)	1.34 (−0.88–3.56)
Trend difference (treatment - control, β_7_ + β_9_ + β_11_)	−0.35 (−4.16–3.46)	−0.23 (−4.10–3.61)	−0.12 (−3.30–3.06)

Note: ^*^ *p* < 0.05, ^**^ *p* < 0.01, ^***^ *p* < 0.001; regression with Newey–West standard errors-lag (1)

## Discussion

This study provides evidence on the impact of a strategic purchasing subsidy reform on the operational efficiency of PHIs in rural Eastern China. Utilizing ITSA, we found that transitioning from passive subsidies (NEPHSP subsidies plus expenditure-revenue deficit subsidies) to a blended payment model (fixed basic salaries plus activity-based RBRVS payments) significantly enhanced efficiency. By channeling about 50% of subsidies through an RVU-based variable pool, the reform converts public money into observable labour time, while the remaining 50% guaranteed block preserves basic income security for all staff—an arrangement that balances efficiency incentives with social protection. Monthly RVUs per employee increased substantially (61.17%) post-reform, demonstrating a sustained positive trend.This aligns with the reform’s core objective and mirrors efficiency gains observed in other contexts implementing activity-based payment, such as the RBRVS system in the U.S. [21].

While the reform successfully increased service volume, we acknowledge the inherent risk of provider-induced demand and quality skimping under activity-based payment [4]. To mitigate these risks, the reform incorporated monthly/yearly RVU caps (see item 13, 14, 17, 22, 23,24 and 26 in Appendix 1), service-specific thresholds, and real-time audits to flag duplicate or medically unnecessary claims. Moreover, NEPHSP services are predominantly preventive with predefined target populations, making them less vulnerable to demand inflation than curative care. Nonetheless, quality metrics were not available in this study; future evaluations should integrate clinical quality indicators and patient-reported outcomes to ensure that volume increases translate into value-based care.

Notably, the efficiency improvement manifested as a gradual acceleration rather than an immediate step-change. This suggests a period of adaptation as PHIs integrated the new incentives, potentially reflecting the complexities of institutional change within established systems [23]. Skepticism among providers towards new payment models, documented elsewhere [24], may also have contributed to this phased response. Another alternative explanation lies in the reform’s timeline. Efficiency had already reached a particular level after 24 months of pre-reform growth, making it challenging to achieve a steeper trajectory over an additional 36 months during the reform period. Our findings contrast with a study in Shaanxi province, which documented a decline in PHC provision by 1.1 to 3.5% for each 1% increment in government subsidy as a proportion of total revenue [12]. This discrepancy underscores the critical role of how subsidies are structured and implemented; strategic purchasing (tying payment to output) appears more effective than passive deficit coverage in driving efficiency within our study context.

The post-COVID slope fell by −1.7 RVUs per staff per month (95% CI −3.1 to −0.24, *p* = 0.012). Although our ITSA design controls for pre-pandemic trends and seasonality, it cannot exclude all concurrent shocks. Under stringent econometric assumptions (no anticipatory effects and no simultaneous disturbances other than the pandemic), ITSA can yield a causal estimate; however, we interpret the 2020 slope change as an association consistent with pandemic disruption rather than definitive proof of causation. This reading aligns with broader evidence that COVID-19 substantially reduced primary-care utilization across China [25] and highlights the vulnerability of efficiency gains to major external shocks, as PHIs struggled to maintain pre-pandemic service levels amid shifting priorities and resource constraints [26].

A crucial finding is the marked heterogeneity in response across PHI types. Remote countryside PHIs showed no significant level change immediately after the reform (β₂ = 10.7, *p* = 0.18), but a significantly steeper post-reform slope (β₃ = +7.6, 95% CI 3.4–11.8, *p* < 0.001). This gradual acceleration, rather than an abrupt jump, suggests that remote institutions required time to adapt to the new incentives. Although remote PHIs exhibited the steepest post-reform RVU growth, this pattern could still reflect unobserved heterogeneity. Thus, we addressed time-invariant heterogeneity with random-effects ITSA. After controlling for institution-specific random intercepts and cluster-robust standard errors, the post-reform slope for remote countryside PHIs remained +9.0 RVUs per staff per month (95% CI 4.7–13.4, *p* < 0.001), virtually identical to the estimate (β₃= +7.6) above. This concordance suggests that unobserved, time-invariant institutional differences do not drive the observed efficiency gains. The trajectory is therefore robust to within-cluster correlation and is consistent with a genuine policy effect rather than a statistical artefact of institutional heterogeneity.

Multi-group ITSA confirmed significant post-reform slope divergence in trends, with a clear gradient: central town/street PHIs < other PHIs < remote countryside PHIs. This gradient reversed during the pandemic, converging as remote PHIs declined most rapidly. This differential impact likely stems from two interrelated factors, one is the subsidy dependence. Smaller, remote PHIs typically rely more heavily on government subsidies for revenue, making them more sensitive to changes in subsidy allocation mechanisms. The other factor is the institutional capacity and agility, as indicated by other researches [27, 28]. While smaller size and limited resources (financial, human, technological) increase vulnerability to shocks [29], they may also allow for greater organizational agility, enabling faster adaptation and response to new incentives like the reform [27]. The counter intuitive finding of greater efficiency gains in smaller/remote PHIs despite lower employee counts supports this notion of heightened responsiveness when appropriately incentivized. However, their inherent resource constraints amplified their vulnerability during the pandemic shock.

A key strength of this study is the use of recent longitudinal data (2015–2022) and robust ITSA methodology, effectively controlling for seasonality, autocorrelation, and the major confounding impact of COVID-19 [20]. Disaggregating results by PHI type provides vital nuance for policymakers. Our findings offer clear policy guidance. Firstly, strategic purchasing works. Blended payment models linking subsidies to activity (RVUs) can effectively enhance PHC efficiency in rural China. Secondly, target support is crucial. While remote and smaller PHIs demonstrated the steepest post-reform slope gains, they also exhibit the highest marginal return on public funds: every CNY 1 million redirected to remote institutions buys 123,000 additional RVUs (versus 75,000 in central-town facilities), a 64% efficiency advantage that persists under random-effects controls. This cost-effectiveness signal implies that scaling-up subsidies towards the smallest, most subsidy-dependent PHIs—while retaining the existing 50% guaranteed-salary block—can amplify output without breaching county fiscal ceilings. Complementary investments could include enhanced remote-area allowances, targeted workforce incentives, and infrastructure upgrades specifically for underserved areas [29], thereby sustaining efficiency gains and bolstering slope-based resilience against future external shocks such as pandemics. Thirdly, further optimization of the activity-based component (RBRVS weights, service scope inclusion) is needed to ensure efficiency gains translate into equitable, high-quality care without unintended consequences like service skimping or cherry-picking. Fourthly, policies must proactively strengthen the resilience of the entire PHC network, particularly its most vulnerable components, to withstand future crises. By demonstrating the potential of strategic purchasing and highlighting the specific needs of remote PHIs, this study provides valuable insights for scaling similar reforms and improving the efficiency and sustainability of primary healthcare in China and comparable settings [30].

## Limitations

Our study has several limitations. Firstly, it was conducted in a single rural county in eastern China, which limits the generalizability of the findings to other regions. Secondly, the absence of an external control county that did not implement the reform prevents us from fully ruling out county-level contemporaneous shocks; future studies should therefore obtain cross-regional data and employ matched or synthetic controls to strengthen causal inference. Thirdly, despite the use of ITSA and adjustment for known confounders such as the COVID-19 pandemic, unmeasured factors—e.g., concurrent local policies, staff turnover, or changes in patient health-seeking behavior—may still bias the results. Fourthly, RVUs serve as a proxy for service volume and intensity but do not capture dimensions of care quality or health outcomes. Future evaluations should incorporate multidimensional indicators. Finally, the quasi-experimental design, while robust for longitudinal policy evaluation, cannot establish causality with the certainty of a randomized controlled trial; complementary methods such as difference-in-differences across multiple counties or staggered adoption designs should be pursued when broader data access is achieved.

## Conclusions

Our interrupted time series analysis demonstrates that China’s strategic subsidy reform, transitioning from passive funding to a blended payment model (fixed basic salaries plus activity-based RBRVS payments), was moderately associated with the long-term operational efficiency of rural PHIs, evidenced by a sustained increase in monthly RVUs per employee.

Critically, this study reveals significant heterogeneity in impact: remote and smaller PHIs exhibited the greatest responsiveness to efficiency incentives introduced by the reform, but also proved the most vulnerable to disruption during the COVID-19 pandemic. This underscores the urgent need for targeted financial and capacity-building support for these institutions to sustain gains and bolster resilience.

This reform provides a valuable model for LMICs seeking to improve primary healthcare efficiency. The evidence suggests that strategic purchasing mechanisms, particularly blended payment models linking subsidies to service output, was associated with performance. However, successful implementation requires careful adaptation to local contexts and proactive measures to protect underserved areas from external shocks. Future reforms should aim to integrate quality and outcome metrics into performance-based payment systems, moving beyond volume-based indicators like RVUs toward a more comprehensive efficiency and quality evaluation framework.

## Electronic supplementary material

Below is the link to the electronic supplementary material.

Supplementary material 1: Appendix 1–the complete RVU catalogue for NEPHSP and complementary medical services, detailing items, service content, data sources, statistical definitions, and quality-control rules

Supplementary material 2: Appendix 2 – autocorrelation diagnostics using the Durbin-Watson test and Cumby-Huizinga general test

Supplementary material 3: Appendix 3 – all Stata do-files for analysis and post-estimation and Appendix 4 – detailed staff numbers and financial subsidies by PHI type before and after the reform (2015–2022, nominal prices).

## Data Availability

The data supporting the findings of this study are available upon request due to privacy concerns and proprietary agreements.

## Abbreviations

CNY: Chinese Yuan
COVID-19: Coronavirus Disease 2019
GPCI: Geographic Practice Cost Index
ICRR: Incremental Cost-RVU Ratio
ITSA: Interrupted Time Series Analysis
LMIC: Low- and Middle-Income Country
NEPHSP: National Essential Public Health Services Package
PHC: Primary Health Care
PHI: Primary Health Institution
RBRVS: Resource-Based Relative Value Scale
RVU: Relative Value Unit
SD: Standard Deviation
