# Supplementary material for: Impact of government subsidy reforms on primary health care efficiency in rural eastern China: an interrupted time series analysis
Source: BMC Health Serv Res. 2026 Jan 7;26:170. doi: 10.1186/s12913-025-13967-0 (PMC12870310; doi:10.1186/s12913-025-13967-0)
Supplement: Supplementary file 1 — Supplementary material 1: Appendix 1–the complete RVU catalogue for NEPHSP and complementary medical services, detailing items, service content, data sources, statistical definitions, and quality-control rules [file 12913_2025_13967_MOESM1_ESM.docx]

**Appendix 1**

**Relative value units catalogue of NEPHSP and complementary compensation for medical services**

**Some explanations**

1. One relative value units (RVU) is the cost of a standard clinic visit including workload (15-min duration), resource consumption, risk, and difficulty.

2. The RVUs of National Essential Public Health Services Package (NEPHSP) services and complementary compensation for medical services are calculated by comparing their cost with that of a standard clinic visit by committees with representatives of Shengzhou county.

3. Notes:* means complementary compensation (named converted RVUs) because of the zero-make-up policy for drugs.

4. RVUs for medical services except outpatient visits and inpatient bed days are [suitable](javascript:;) [for](javascript:;) the circumstance without their fee-for-service items.

5. NEPHSP services are classified as either individual or group interventions.For individual services, total RVUs equal the RVU weight per visit multiplied by the number of visits delivered; for group services, total RVUs equal the per-capita RVU weight multiplied by the enrolled population.

| **Categories** | **Items** | RVUs | **Service contents** | **Data source** | **Calculation/**quality control |
| --- | --- | --- | --- | --- | --- |
| Medical services | 1. Outpatient visits (per visit) | 0.4* | To provide medical history inquiry, physical examination, medical treatment, health promotion, medical records, etc. | Hospital information system (HIS) | 1. In accordance with the charging system by registration.  2. One doctor's visit is counted as one visit within one day (0:00 to 24:00). |
|  | 2. Home visits (per visit) | 3 | To visit the patient's residence to provide relevant medical services. Its requirement is the same as for outpatient visit. | HIS | 1. In accordance with the charging system by home visit fee and family sickbed visit.  2. One doctor's visit is counted as one visit within one day (0:00 to 24:00). |
|  | 3. Inpatient bed days (per bed day) | 2* | To provide disease diagnosis and treatment, medical examination, health promotion, medical records, etc. | HIS | 1. Occupied bed days of the inpatients.  2. In accordance with the charging system. |
|  | 4. Family sickbeds (per bed) | 4 | To provide needs assessment, agreement signature, preparations for family sickbed, personalized treatment plan, health promotion, medical records, etc. | HIS | 1. In accordance with the charging system.  2. Counted as one bed from admission to discharge. |
|  | 5. Medical referral visits (per service) | 0.8 | To refer service upward or downward between hospitals and primary health institutions. | Medical referral system | Completing the whole process. |
|  | 6. Visits to discharged patients from hospital (per visit) | 2 | To provide guidance for rehabilitation, pharmacy practice, nutrition and health behavior, health promotion, etc. | Electronic health record system (EHR) | Calculated as one visit for each discharged patient. |
| NEPHSP | 7. Establishing new health records (per enrolled citizen) | 3 | To set up EHR including personal basic information, physical examination, public health service records, health evaluation, etc. | EHR | Calculated according to newly established health records each year. |
|  | 8. Updating health records (per enrolled citizen) | 0.2 | To dynamically update health records according to the requirements. | EHR | Those unqualified health records should be eliminated.  1. Wrong ID number.  2. Blank or incorrect entries ≥2 in personal basic information.  3. Blank or incorrect entries ≥3 or lack of health evaluation in physical examination. |
|  | 9. Immunization (per inoculation) | 1.5 | To inform children for vaccination, pre-inspect, register, vaccinate, observation, and management service such as statistical analysis, under-reporting investigation, abnormal vaccination reaction treatment, etc. | EHR | The inoculated children aged 0-7 for planned vaccination. |
|  | 10. Establishing vaccination certificates (per child aged 0-6 years) | 0.5 | To establish vaccination certificates for children aged 0-6 years old who have lived in the district for more than 6 months, and checking them every 6 months. | Vaccination management system | Newly established vaccination certificate. |
|  | 11. Home visits to newborn infants (per visit) | 3 | To provide inquiry of neonatal birth condition, vaccination, newborn disease screening, feeding, sleeping, urine and feces, jaundice, umbilical cord, etc. To provide temperature measurement, cardiopulmonary auscultation, abdominal palpation and other examinations, health guidance, health record entry, etc. | Maternal and child health information system (MCHIS) | In accordance with the newborn visit records. |
| NEPHSP | 12. Establishing newborn health care booklet (per enrolled baby) | 0.5 | To establish a booklet including consultation, physical examination, development evaluation, health guidance, health record entry, etc. | MCHIS | In accordance with the newborn visit records. |
|  | 13. Health management of children aged 0-3 (per visit) | 2.5 | To provide eight physical examinations and developmental assessment at 3, 6, 8, 12, 18, 24, 30, and 36 months old by inquiring about feeding and illness. To provide routine blood tests at 6-8, 18, and 30 months old. To provide hearing screening at 6, 12, 24, and 36 months old. | MCHIS | According to the number of health examination records of children aged 0-3. The cap of visits for group aged 0-1, 1-2 and 2-3 is four, two and one respectively. |
|  | 14. Health management of children aged 4-6 (per visit) | 2 | To provide management once a year including inquiry about diet, illness, physical examination, blood routine examination, visual screening and development assessment, health guidance, etc. | MCHIS | According to the statistics of the health examination records of children aged 4-6. The cap is one time per year. |
|  | 15. Management of high-risk infants and children with nutritional diseases (per visit) | 2 | To screen out high-risk infants and children with nutritional diseases, increase the number of follow-up visits, give targeted interventions and guidance, transfer to higher institutions when necessary, evaluate cases when growth and development are normal, symptoms and signs are reduced or disappeared. | MCHIS | According to the number of follow-up management records of high-risk children with nutritional diseases. |
| NEPHSP | 16. Health management for pregnant women before 12 weeks (per enrolled woman) | 8 | To establish maternal health care booklets for pregnant women before 12 weeks of pregnancy, carry out inquiry, physical examination, laboratory and auxiliary examination, give health guidance such as health, psychology, nutrition, prenatal screening, and avoidance of teratogenic factors. | MCHIS | According to the number of newly established maternal health records. |
|  | 17. Antenatal examination (per visit) | 1.5 | To provide examination once every 16-20, 21-24, 28-36, 37-40 weeks of gestation, including general physical examination, obstetric examination and necessary auxiliary examinations, prenatal screening, prenatal diabetes screening, and screening of high-risk pregnant women according to regulations. | MCHIS | The cap of prenatal examination visits is the number of pregnant women × 8. |
|  | 18. Postpartum visits (per visit) | 3 | To provide maternal home visits within 3 to 7 days after delivery including recording blood pressure and body temperature ,breast and uterus involution , guidance of hygiene, nutrition, rehabilitation, breastfeeding and neonatal care. Recording them in the maternal health information system. | MCHIS | According to the postpartum visits. |
|  | 19. Postpartum 42-day check (per enrolled woman) | 1 | To conduct inquiries, measure blood pressure, body temperature and weight, check breast and uterus involution, give health guidance such as postpartum rehabilitation. Data are recorded in the maternal health information system. | MCHIS | According to the record of postpartum 42-day check. |
| NEPHSP | 20. Management of high-risk pregnant women (per enrolled woman) | 4 | To assess, report, and follow-up high-risk pregnant women identified by screening and refer them when necessary. The high-risk factors were reviewed and concluded at the postpartum 42-day check. | MCHIS | According to the number of high-risk pregnant women. |
|  | 21. Health management of the elderly (per enrolled elderly) | 7 | To provide health management services once a year for the elderly over 60 including lifestyle inquiries, physical examinations, supplementary examinations, health assessment, and health guidance. | EHR | According to the number of the elderly who have established the record. |
|  | 22. Health management of hypertension (per visit) | 2.5 | To measure the blood pressure of first-visit patients over 35 years. To find hypertensive patients through physical examination, follow-up, etc. To implement graded management of hypertension, Level 1: health check-up once a year, 4 times follow-up a year; Level 2: plus 2 times follow-up a year; Level 3: plus 6 times a year. Data are recorded in the maternal health information system. | EHR | According to the number of patients with hypertension under standardized management. The number of people whose follow-up records are not qualified (blank, missing and wrong items are more than or equal to 2 items, blood pressure value is not filled in, and blood pressure is not controlled for two consecutive times, etc.) will be excluded. The number of first-class, second-class and third-class follow-up was counted respectively. The cap of visits is limited by the number patients with first-, second-, or third-level management (4, 6, or 12 times, respectively). |
| NEPHSP | 23. Health management of diabetes(per visit) | 2.75 | To identify diabetes mellitus patients through outpatient visits, physical examination, follow-up, etc. To implement graded management: health check-up once a year, follow-up routine management once every 3 months, strengthened management once a month. | EHR | According to the number of patients with hypertension under standardized management. The number of people whose follow-up records are not qualified (blank, missing and wrong items are more than or equal to 2 items, blood sugar value is not filled in, blood sugar is not controlled for two consecutive times) will be excluded. The numbers of follow-up visits for routine and intensive management were counted. The cap of visits is limited by the number patients under regular management (4 times) and strengthening management (12 times). |
|  | 24. Management of patients with severe mental disorders (per visit) | 3 | To provide regular community investigation, refer suspected patients for diagnosis, provide health management including follow-up, physical examination, evaluation, intervention, two-way referral, and emergency treatment. To implement graded management:   - Risk level 3-5: physical examination once a year for patients with unstable condition, transfer treatment, and follow-up for outpatients. - Risk level 1-2: follow-up after treatment, regular follow-up every 3 months. - Risk level 0: follow-up every 3 months. | Management system for severe mental disorders | According to the number of patients with severe mental disorders under standardized management. Patients whose follow-up records are not qualified (blank items, blank items, wrong items >4) will be excluded. The numbers of follow-up visits for routine management and intensive management were counted. The cap of visits is limited by the number patients in level 3-5, level 1-2, and level 0 (12, 6, and 4 visits, respectively). |
|  | 25.Traditional Chinese Medicine (TCM) management for the elderly aged 65+ (per enrolled elderly) | 2 | To provide service once a year including TCM constitution identification and TCM health care guidance, physical identification etc. | EHR | According to the number of elderly people aged 65+ who received TCM services. |
|  | 26. TCM management for children (per visit) | 0.4 | To provide TCM health care guidance to parents of children aged 6, 12, 18, 24, 30, and 36 months including TCM diet, abdominal moistening, chiropractic, acupoint massage method guidance, etc. | MCHIS | According to the number of children in MCHIS. The visit cap for groups aged  0-1, 1-2, and 2-3 are 1, 2, and 3 times, respectively. |
| NEPHSP | 27. Report on infectious diseases and public health emergencies (per permanent resident) | 0.08 | To assist in risk investigation, collection, and provision of risk information on infectious diseases and public health emergencies; to standardize the registration and reporting of information on infectious diseases and public health emergencies, etc. | None | According to the number of permanent residents. |
|  | 28. Health education (per permanent resident) | 0.2 | To provide health education materials, each institution needs to provide 12 kinds of printed material and 6 kinds of image material at least every year; to set up health education propaganda boards at least 6 times a year; to carry out public health consultation services at least 9 times a year; to provide health knowledge lectures at least 12 times a year; to provide individualized health education in combination with outpatient clinics and visits. | None | According to the number of permanent residents. |
|  | 29. Health supervision and co-management (per permanent resident) | 0.03 | To provide report on food safety, occupational diseases, drinking water safety, school hygiene, illegal medical practice and other incidents; to inspect drinking water hygiene, school hygiene, and illegal medical practice four times a year. | None | According to the number of permanent residents. |
