# Supplementary material for: Impact of government subsidy reforms on primary health care efficiency in rural eastern China: an interrupted time series analysis
Source: BMC Health Serv Res. 2026 Jan 7;26:170. doi: 10.1186/s12913-025-13967-0 (PMC12870310; doi:10.1186/s12913-025-13967-0)
Supplement: Supplementary file 2 — Supplementary material 2: Appendix 2 – autocorrelation diagnostics using the Durbin-Watson test and Cumby-Huizinga general test [file 12913_2025_13967_MOESM2_ESM.docx]

**Appendix 2**

**Table.** Autocorrelation test of monthly relative-value units per employee.

| **Category** | **Durbin–Watson test** | **Cumby–Huizinga test(χ^2^, df=1)** | | |
| --- | --- | --- | --- | --- |
|  |  | **Lag(1)** | **Lag(2)** | **Lag(3)** |
| All PHIs | 1.369 | 5.295* | 2.364 | 0.320 |
| Central town and street PHIs | 1.632 | 2.601 | 0.337 | 0.232 |
| Remote countryside PHIs | 0.786 | 21.219^***^ | 8.195^***^ | 3.036 |
| Other township PHIs | 1.020 | 13.897^***^ | 3.620 | 0.990 |

Note: ^*^ *P* <0.05, ^**^ *P* <0.01, ^***^ *P*<0.001; PHI = primary health institution.
