# Supplementary material for: Impact of government subsidy reforms on primary health care efficiency in rural eastern China: an interrupted time series analysis
Source: BMC Health Serv Res. 2026 Jan 7;26:170. doi: 10.1186/s12913-025-13967-0 (PMC12870310; doi:10.1186/s12913-025-13967-0)
Supplement: Supplementary file 3 — Supplementary material 3: Appendix 3 – all Stata do-files for analysis and post-estimation and Appendix 4 – detailed staff numbers and financial subsidies by PHI type before and after the reform (2015–2022, nominal prices). [file 12913_2025_13967_MOESM3_ESM.docx]

**Appendix 3. STATA-do-file**

**Appendix 4. Staff and subsidies by PHI type for primary health institutions before and after the reform**

**Appendix 3. STATA-do-file**

STATA-do-file

* Single-group ITSA for all PHIs

clear

use All PHIs.dta, clear

graph twoway scatter rbrvsperstaff time

regr rbrvsperstaff time intervention post

*Check the residuals by plotting against time

predict res, r

twoway (scatter res time)(lowess res time),yline(0)

drop res

*Further check for autocorrelation by examining the autocorrelation

estat dwatson

rvfplot

actest, lag(3) robust

*single-group itsa compares the level and trend before and after two interventions with autocorrelation treatment lag(1)

itsa rbrvsperstaff, single trperiod(25;61) lag(1) replace posttrend figure

gen degrees=(time/12)*360 //seasonal patter is adjusted by fitting Fourier terms in the regression model

fourier degrees, n(2) // help fourier, then install circular from http://fmwww.bc.edu/RePEc/bocode/c

glm rbrvsperstaff cos* sin* _t _x25 _x_t25 _x61 _x_t61, family(gaussian) scale(x2)

// family(gaussian) link is the canonical link for the family

// scale(x2) is assumed for the continuous distributions (Gaussian, gamma, and inverse Gaussian).

predict pred2, nooffset // predicted mean rbrvsperstaff

*Twoway scatter of actual values, seasonality(by fitting Fourier terms) and fitted values(glm model)

twoway (scatter rbrvsperstaff time) (line pred2 time, lcolor(red)) (line _s_rbrvsperstaff_pred time, lcolor(red) lpattern(dash)), title("All primary health institutions") ///

ytitle(RBRVS per staff) ylabel(#5, labsize(small) angle(horizontal)) ///

xtick(0.5(12)96.5) xlabel(1"2015Jan" 13"2016Jan" 25"2017Jan" 37"2018Jan" 49"2019Jan" 61"2020Jan" 73"2021Jan" 85"2022Jan" 97"2023Jan", noticks labsize(small)) xtitle(Months) ///

xline(24.5 60.5)

drop pred2

* POINT ESTIMATE: post-reform slope β₁ + β₃

* (using Newey-West SEs with lag 1, identical to the glm specification)

newey rbrvsperstaff cos* sin* _t _x25 _x_t25 _x61 _x_t61, lag(1)

lincom _t + _x_t25 // β₁ + β₃ → post-reform slope

scalar post_slope = r(estimate)

scalar post_se = r(se)

scalar post_p = r(p)

* ---------------------------------------------------------------

* 1. RE-ESTIMATE FULL MODEL (identical covariates & autocorrection)

* ---------------------------------------------------------------

newey rbrvsperstaff cos* sin* _t _x25 _x_t25 _x61 _x_t61, lag(1)

* ---------------------------------------------------------------

* 2. POINT ESTIMATE: POST-COVID TREND β₁ + β₃ + β₅

* (reform = _x25, COVID = _x61)

* ---------------------------------------------------------------

lincom _t + _x_t25 + _x_t61

* returns r(estimate), r(se), r(p)

local beta1_p3_p5 = r(estimate)

local se_nevey = r(se)

local p_val = r(p)

* Single-group ITSA for category1(Central town and street PHIs)

use Sub-category.dta, clear

drop if category==2 // Other PHIs

drop if category==3 // Remote countryside PHIs

graph twoway scatter rbrvsperstaff time

regr rbrvsperstaff time _x25 _x_t25

*Check the residuals by plotting against time

predict res, r

twoway (scatter res time)(lowess res time),yline(0)

drop res

*Further check for autocorrelation by examining the autocorrelation

estat dwatson

rvfplot

actest, lag(3) robust

*single-group itsa compares the level and trend before and after two interventions without autocorrelation

itsa rbrvsperstaff, single trperiod(25;61) replace posttrend figure

gen degrees=(time/12)*360 //seasonal patter is adjusted by fitting Fourier terms in the regression model

fourier degrees, n(2)

glm rbrvsperstaff cos* sin* _t _x25 _x_t25 _x61 _x_t61, family(gaussian) scale(x2)

// family(gaussian) link is the canonical link for the family

// scale(x2) is assumed for the continuous distributions (Gaussian, gamma, and inverse Gaussian).

predict pred2, nooffset

twoway (scatter rbrvsperstaff time) (line pred2 time, lcolor(red)) (line _s_rbrvsperstaff_pred time, lcolor(red) lpattern(dash)), title("Central town and street PHIs") ///

ytitle(RBRVS per staff) ylabel(#5, labsize(small) angle(horizontal)) ///

xtick(0.5(12)96.5) xlabel(1"2015Jan" 13"2016Jan" 25"2017Jan" 37"2018Jan" 49"2019Jan" 61"2020Jan" 73"2021Jan" 85"2022Jan" 97"2023Jan", noticks labsize(small)) xtitle(Months) ///

xline(24.5 60.5)

drop pred2

* ------------------------------------------------------------------

* 1. RE-ESTIMATE FULL MODEL (identical covariates & autocorrection)

* ------------------------------------------------------------------

newey rbrvsperstaff cos* sin* _t _x25 _x_t25 _x61 _x_t61, lag(1)

* ------------------------------------------------------------------

* 2. POINT ESTIMATE: POST-REFORM TREND β₁ + β₃

* ------------------------------------------------------------------

lincom _t + _x_t25

local slopeRef = r(estimate)

local seRef = r(se)

local pRef = r(p)

* ------------------------------------------------------------------

* 3. POINT ESTIMATE: POST-COVID TREND β₁ + β₃ + β₅

* ------------------------------------------------------------------

lincom _t + _x_t25 + _x_t61

local slopeCovid = r(estimate)

local seCovid = r(se)

local pCovid = r(p)

*Single-group ITSA for category2(Other PHIs)

use Sub-category.dta, clear

drop if category==1 //Central town and street PHIs

drop if category==3 //Remote countryside PHIs

tsset time

graph twoway scatter rbrvsperstaff time

regr rbrvsperstaff time _x25 _x_t25

*Check the residuals by plotting against time

predict res, r

twoway (scatter res time)(lowess res time),yline(0)

drop res

*Further check for autocorrelation by examining the autocorrelation

estat dwatson

rvfplot

actest, lag(3) robust

*single-group itsa compares the level and trend before and after two interventions with autocorrelation treatment lag(1)

itsa rbrvsperstaff, single trperiod(25;61) lag(1) replace posttrend figure

gen degrees=(time/12)*360 //seasonal patter is adjusted by fitting Fourier terms in the regression model

fourier degrees, n(2)

glm rbrvsperstaff cos* sin* _t _x25 _x_t25 _x61 _x_t61, family(gaussian) scale(x2)

// family(gaussian) link is the canonical link for the family

// scale(x2) is assumed for the continuous distributions (Gaussian, gamma, and inverse Gaussian).

predict pred2, nooffset

twoway (scatter rbrvsperstaff time) (line pred2 time, lcolor(red)) (line _s_rbrvsperstaff_pred time, lcolor(red) lpattern(dash)), title("Other PHIs") ///

ytitle(RBRVS per staff) ylabel(#5, labsize(small) angle(horizontal)) ///

xtick(0.5(12)96.5) xlabel(1"2015Jan" 13"2016Jan" 25"2017Jan" 37"2018Jan" 49"2019Jan" 61"2020Jan" 73"2021Jan" 85"2022Jan" 97"2023Jan", noticks labsize(small)) xtitle(Months) ///

xline(24.5 60.5)

drop pred2

* ------------------------------------------------------------------

* 1. RE-ESTIMATE FULL MODEL (identical covariates & autocorrection)

* ------------------------------------------------------------------

newey rbrvsperstaff cos* sin* _t _x25 _x_t25 _x61 _x_t61, lag(1)

* ------------------------------------------------------------------

* 2. POINT ESTIMATE: POST-REFORM TREND β₁ + β₃

* ------------------------------------------------------------------

lincom _t + _x_t25

local slopeRef = r(estimate)

local seRef = r(se)

local pRef = r(p)

* ------------------------------------------------------------------

* 3. POINT ESTIMATE: POST-COVID TREND β₁ + β₃ + β₅

* ------------------------------------------------------------------

lincom _t + _x_t25 + _x_t61

local slopeCovid = r(estimate)

local seCovid = r(se)

local pCovid = r(p)

*Single-group ITSA for category3 (Remote countryside PHIs)

use Sub-category.dta, clear

drop if category==1

drop if category==2

tsset time

graph twoway scatter rbrvsperstaff time

regr rbrvsperstaff time _x25 _x_t25

*Check the residuals by plotting against time

predict res, r

twoway (scatter res time)(lowess res time),yline(0)

drop res

*Further check for autocorrelation by examining the autocorrelation

estat dwatson

rvfplot

actest, lag(3) robust

*single-group itsa compares the level and trend before and after two interventions with autocorrelation treatment lag(2)

itsa rbrvsperstaff, single trperiod(25;61) lag(2) replace posttrend figure

gen degrees=(time/12)*360 //seasonal patter is adjusted by fitting Fourier terms in the regression model

fourier degrees, n(2)

glm rbrvsperstaff cos* sin* _t _x25 _x_t25 _x61 _x_t61, family(gaussian) scale(x2)

// family(gaussian) link is the canonical link for the family

// scale(x2) is assumed for the continuous distributions (Gaussian, gamma, and inverse Gaussian).

predict pred2, nooffset

twoway (scatter rbrvsperstaff time) (line pred2 time, lcolor(red)) (line _s_rbrvsperstaff_pred time, lcolor(red) lpattern(dash)), title("Other PHIs") ///

ytitle(RBRVS per staff) ylabel(#5, labsize(small) angle(horizontal)) ///

xtick(0.5(12)96.5) xlabel(1"2015Jan" 13"2016Jan" 25"2017Jan" 37"2018Jan" 49"2019Jan" 61"2020Jan" 73"2021Jan" 85"2022Jan" 97"2023Jan", noticks labsize(small)) xtitle(Months) ///

xline(24.5 60.5)

drop pred2

* ------------------------------------------------------------------

* 1. RE-ESTIMATE FULL MODEL (identical covariates & autocorrection)

* ------------------------------------------------------------------

newey rbrvsperstaff cos* sin* _t _x25 _x_t25 _x61 _x_t61, lag(1)

* ------------------------------------------------------------------

* 2. POINT ESTIMATE: POST-REFORM TREND β₁ + β₃

* ------------------------------------------------------------------

lincom _t + _x_t25

local slopeRef = r(estimate)

local seRef = r(se)

local pRef = r(p)

* ------------------------------------------------------------------

* 3. POINT ESTIMATE: POST-COVID TREND β₁ + β₃ + β₅

* ------------------------------------------------------------------

lincom _t + _x_t25 + _x_t61

local slopeCovid = r(estimate)

local seCovid = r(se)

local pCovid = r(p)

*mutiple-group itsa rbrvsperstaff

use Sub-category.dta, clear

tsset category time

itsa rbrvsperstaff, treat(1) contid(2) trperiod(25;61) replace lag(1) posttrend figure

drop if category==1

drop if category==3

gen degrees=(time/12)*360 //seasonal patter is adjusted by fitting Fourier terms in the regression model

fourier degrees, n(2)

glm rbrvsperstaff cos* sin* _t _x25 _x_t25 _x61 _x_t61, family(gaussian) scale(x2)

// family(gaussian) link is the canonical link for the family

// scale(x2) is assumed for the continuous distributions (Gaussian, gamma, and inverse Gaussian).

predict pred2, nooffset

addplot:(line pred2 time, lcolor(red))

addplot:,legend(off)

clear

use Sub-category.dta, clear

drop if category==2

drop if category==3

gen degrees=(time/12)*360

fourier degrees, n(2)

glm rbrvsperstaff cos* sin* _t _x25 _x_t25 _x61 _x_t61, family(gaussian) scale(x2)

predict pred2, nooffset

addplot:(line pred2 time, lcolor(black))

addplot:,legend(off)

clear

graph save "Graph" "Graph1.gph"

use Sub-category.dta, clear

drop if category==1

drop if category==2

tsset time

itsa rbrvsperstaff, single trperiod(25 61) replace posttrend figure

gen degrees=(time/12)*360

fourier degrees, n(2)

glm rbrvsperstaff cos* sin* _t _x25 _x_t25 _x61 _x_t61, family(gaussian) scale(x2)

predict pred2, nooffset

twoway (scatter rbrvsperstaff time) (line pred2 time, lcolor(red)) (line _s_rbrvsperstaff_pred time, lcolor(red) lpattern(dash)), title("") ///

ytitle() ylabel(150 (50) 550, labsize(small) angle(horizontal)) ///

xtick(0.5(12)96.5) xlabel(1"2015Jan" 13"2016Jan" 25"2017Jan" 37"2018Jan" 49"2019Jan" 61"2020Jan" 73"2021Jan" 85"2022Jan" 97"2023Jan", noticks labsize(small)) xtitle(Months) ///

xline(24.5 60.5)

graph use Graph1.gph

addplot:(scatter rbrvsperstaff time,msymbol(x) mcolor(blue) msize(small))(line pred2 time, lcolor(blue))(line _s_rbrvsperstaff_pred time, lcolor(blue) lpattern(dash))

addplot:,legend(off)

Appendix 4.

Staff and subsidies by PHI type for primary health institutions before and after the reform

| **Facility type** | **Items** | **Before reform**  **(24 months)** | | |  | **After the pilot reform**  **(36 months)** | | | |  | **After COVID-19 pandemic**  **(36 months)** | | | |
| --- | --- | --- | --- | --- | --- | --- | --- | --- | --- | --- | --- | --- | --- | --- |
|  |  | **2015** | **2016** | **Mean** |  | **2017** | **2018** | **2019** | **Mean** |  | **2020** | **2021** | **2022** | **Mean** |
| Central town and street PHIs(n=9) | Total employees | 1215 | 1238 | 1226.5 |  | 1163 | 1125 | 1108 | 1132 |  | 1100 | 1143 | 1173 | 1138.67 |
|  | Total financial subsidy (CNY million) | 85.23 | 86.56 | 85.90 |  | 119.48 | 130.32 | 132.87 | 127.56 |  | 133.07 | 133.31 | 190.56 | 152.31 |
| Remote countryside PHIs(n=6) | Total employees | 234 | 240 | 237 |  | 257 | 215 | 218 | 230 |  | 213 | 224 | 229 | 222 |
|  | Total financial subsidy (CNY million) | 23.2 | 22.95 | 23.08 |  | 25.96 | 34.44 | 35.32 | 31.91 |  | 34.18 | 33.31 | 42.27 | 36.59 |
| Other PHIs(n=6) | Total employees | 60 | 61 | 60.5 |  | 64 | 41 | 42 | 49 |  | 43 | 42 | 44 | 43 |
|  | Total financial subsidy (CNY million) | 4.83 | 5.3 | 5.065 |  | 7.82 | 8.14 | 8.46 | 8.14 |  | 8.8 | 8.49 | 9.63 | 8.97 |
| All(n=21) | Total employees | 1509 | 1539 | 1524 |  | 1484 | 1381 | 1368 | 1411 |  | 1356 | 1409 | 1446 | 1404 |
|  | Total financial subsidy (CNY million) | 113.26 | 114.81 | 114.04 |  | 153.26 | 172.9 | 176.65 | 167.60 |  | 176.05 | 175.12 | 242.46 | 197.88 |
